# Combating antimicrobial resistance through multimodal data via artificial intelligence

**DOI:** 10.1016/j.onehlt.2026.101573

**Published:** 2026-09-20

**Authors:** Qi Xia, Jiayang Li, Wenqi Wu, Jiajie Wang, Zhitao Zhou, Meilin Wu, Mingjie Qiu, Li Xu, Jianan Ren, Xiuwen Wu

**Affiliations:** a School of Medicine, Southeast University, Nanjing, China; b Research Institute of General Surgery, Jinling Hospital, School of Medicine, Southeast University, Nanjing, China; c Research Institute of General Surgery, Jinling Hospital, the Affiliated Hospital of Medical School, Nanjing University, Nanjing, China; d Research Institute of General Surgery, the Jinling School of Clinical Medicine, Nanjing Medical University, Nanjing, China

**Keywords:** Antimicrobial resistance, Artificial intelligence, Large language model, Drug discovery, Machine learning, One Health

## Abstract

Antimicrobial resistance (AMR) poses a critical global health challenge, leading to substantial mortality and economic losses without science-based interventions. Conventional approaches to combating AMR have important limitations in addressing this escalating threat. Recent advances in AI, including deep learning, large language models, and protein language models, provide new opportunities to integrate multimodal data for AMR research. However, evidence remains uneven, with many approaches limited to computational benchmarking rather than real-world validation. Here, we critically review AI applications in AMR, focusing on rapid diagnosis, therapeutic discovery, and clinical decision support. AI-based diagnostic models can identify resistant pathogens and predict resistance profiles, but most lack prospective clinical evaluation. AI-assisted drug discovery and alternative therapies have generated promising candidates, although most remain at computational, in vitro, or early preclinical stages. Large language models may support decision-making, but their clinical use remains preliminary. Successful translation of AI into clinical and One Health practice will require representative datasets, independent validation, prospective evaluation, and robust governance.

## Highlights

• Artificial intelligence enables rapid diagnosis from molecular and physical data.
• Generative tools support antibiotic, peptide, phage, and inhibitor discovery.
• AI and language models may support clinical decisions and One Health surveillance.
• Translation requires representative data, robust validation, and responsible governance.

## Graphical abstract

**Figure:**
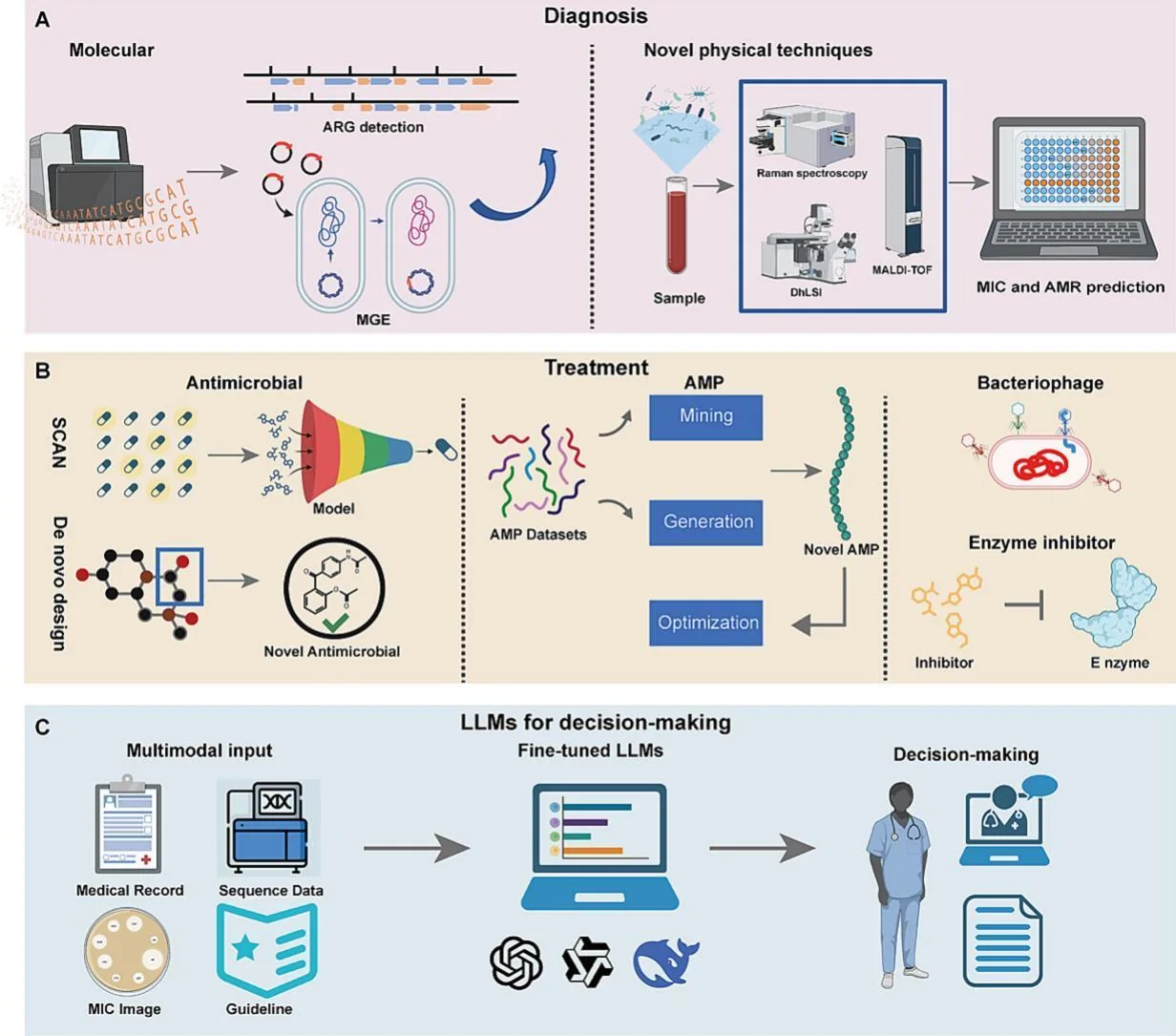

## 1 Introduction

Antimicrobial resistance (AMR) is the evolving process by which bacteria, viruses, fungi, and parasites develop resistance to medicines previously effective against them. According to the most recent Global Burden of Disease analysis, bacterial AMR was directly responsible for 1.14 million global deaths in 2021 and contributed to a total of 4.71 million deaths. Without effective intervention, drug-resistant bacterial infections could lead to 8.22 million deaths globally in 2050, with a particularly heavy toll expected in South Asia and Latin America and the Caribbean [1].

The emergence of AI technologies, particularly foundation models such as large language models (LLMs) and protein language models (PLMs), has greatly expanded the role of AI in biology [2]. Across this hierarchy, methods range from conventional machine learning to deep learning, including convolutional neural networks (CNNs) and Transformer-based architectures. Within the Transformer family, BERT-derived models, protein language models, and LLMs have become especially influential in biological and biomedical research. Protein language models such as ESM-2, together with structure-prediction systems such as AlphaFold3, are reshaping protein research methodologies [3]. In the AMR field, these approaches support a broad range of tasks, including resistance prediction, diagnosis, antimicrobial discovery, and decision support [4]. To make these relationships more explicit, we provide an introductory hierarchical framework (Fig. 1A).

**Fig. 1:**
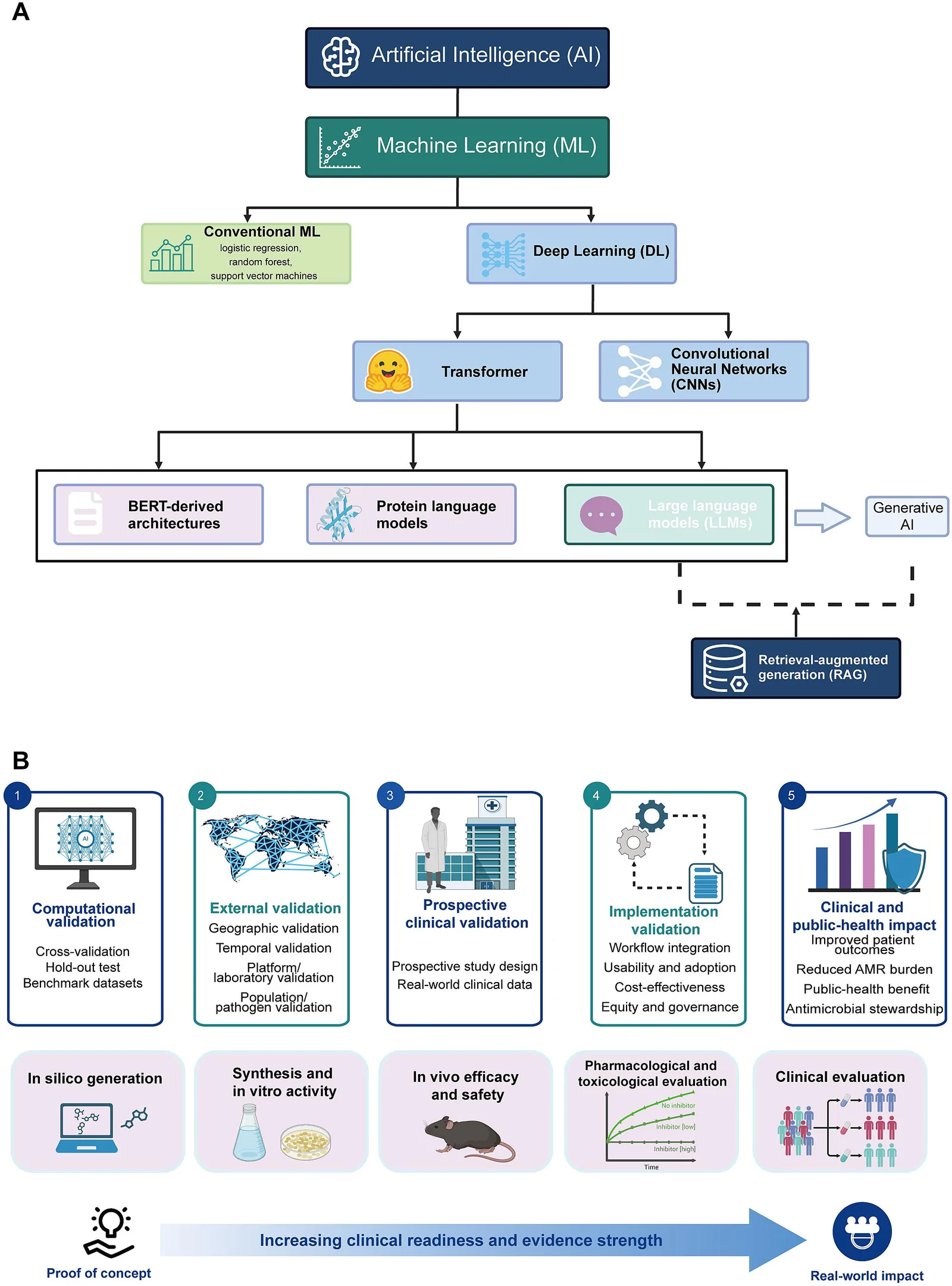
The figure illustrates a hierarchical AI framework and its translational framework from proof of concept to real-world impact in AMR research. (A) Machine learning comprises conventional methods, such as logistic regression, random forests, and support vector machines, as well as deep learning. Deep-learning architectures include CNNs and Transformers, with the latter encompassing BERT-derived architectures, protein language models, and LLMs. Generative AI enables new content generation, whereas RAG augments model outputs with information retrieved from external knowledge sources. (B) AI technologies require progressive evidence generation, moving from computational and external validation to prospective evaluation, implementation, and demonstrated clinical or public-health benefit. Similarly, AI-assisted antimicrobial discovery requires validation beyond in silico prediction, including experimental, preclinical, and clinical assessment. CNN, convolutional neural network; LLM, large language model; ML, machine learning; RAG, retrieval-augmented generation.

Traditional approaches to combating AMR face three major challenges spanning diagnosis, treatment, and decision-making. Empiric antibiotic prescribing, commonly employed before time-consuming diagnostic results are available, can lead to non-targeted antibiotic use. Furthermore, conventional drug discovery struggles to keep pace with the rapid evolution of resistant pathogens. Decision-making for clinicians and policy formulation by governments are also complicated by regional developmental disparities.

In this review, we investigate the application of AI to address these challenges, leveraging the most advanced techniques such as complex models and high-performance LLMs. AI-driven diagnostics can reduce reliance on empiric therapy by enabling rapid pathogen identification and resistance prediction. In treatment, AI methods, including protein language models like ESM-2, expedite the development of novel antibiotics and alternative therapies. For decision-making, LLMs integrate multimodal data to support personalized treatment choices and policy formulation. However, strong predictive performance alone does not establish the clinical value of AI applications in AMR. We therefore use a five-stage translational framework to distinguish proof-of-concept performance from clinical utility (Fig. 1B). The stages comprise computational validation, external validation, prospective clinical validation, implementation evaluation, and demonstrated clinical or public-health impact. The distinctive contribution of this review is to connect traditionally separated AI applications across AMR within a unified framework based on data modality and translational stage from a One Health perspective. Rather than considering these applications in isolation, we examine how multimodal data are processed by AI to support resistance detection, antimicrobial discovery, clinical decision-making, and surveillance. Particular attention is given to foundation models and language-model-based approaches that bridge sequence interpretation, therapeutic generation, and knowledge-guided decision support. This cross-scale perspective enables us to assess not only computational advances but also the evidence required for their clinical and public-health translation.

## 2 Methods

This article was designed as a narrative review summarizing recent applications of AI in addressing AMR. We searched PubMed, Web of Science, and Scopus for English-language articles published through December 2025. During manuscript revision, we conducted a targeted supplementary search and incorporated relevant studies published online through August 2026. Search terms related to AMR were combined with terms related to AI and its applications in diagnosis, drug discovery, antimicrobial peptide development, phage therapy, clinical decision support, antimicrobial stewardship, surveillance, policy, and One Health. For One Health-related applications, studies involving human, animal, food-production, and environmental settings were considered.

Studies were included if they described an AI application directly relevant to AMR and provided sufficient information on the data, methods, and outcomes. Studies that referred only incidentally to AI or AMR, provided insufficient methodological information, or duplicated previously reported findings were excluded. Given the heterogeneity of the included studies, the evidence was synthesized narratively.

## 3 AI-enhanced diagnosis of antimicrobial resistance

To combat antimicrobial resistance (AMR), diagnostics—particularly rapid diagnostics—play a pivotal role. Conventional phenotypic methods, such as antimicrobial susceptibility testing (AST) and MIC determination, remain the gold standard. However, these approaches are time-consuming due to reliance on pure cultures and provide limited insight into the mechanisms underlying resistance.

Molecular diagnostics focus on genetic, transcriptional, and metabolic data. Polymerase chain reaction (PCR) is widely adopted in clinical detection. Advances in next-generation sequencing (NGS) and third-generation sequencing technologies have broadened diagnostic capabilities for faster results. Whole-genome sequencing (WGS) enables comprehensive detection of antimicrobial resistance genes (ARGs), genetic mutation and mechanistic interpretation from a genetic perspective [5]. Metagenomic data can identify diverse pathogenic taxa, ARGs, and horizontal gene transfer mechanisms via mobile genetic elements (MGEs).

Beyond phenotypic and molecular diagnostics, novel techniques leveraging physical properties have emerged. Matrix-assisted laser desorption/ionization time-of-flight (MALDI-TOF) mass spectrometry, Raman spectroscopy, and dynamic holographic laser speckle imaging represent innovative diagnostic tools enhancing rapid AMR detection.

AI with established diagnostic methodologies significantly enhances efficiency and accuracy in antimicrobial resistance detection. Advancements in AI, particularly DL, have augmented conventional approaches. CNNs, attention-based architectures, and BERT-derived models can predict putative ARGs with limited sequence similarity to known resistance genes, thereby expanding the pool of candidates for experimental validation. Concurrently, LLMs support clinical decision-making by processing multimodal inputs, including electronic medical records, sequence data, medical images, lab results, and clinical guidelines.

### 3.1 AI for rapid molecular diagnosis

The main determinants of AMR are the ARGs. The traditional method is based on alignment with ARG datasets like CARD [6], ARDB [7], ResFinder [8] and AMRFinder [9]. The limitation of methods based on alignment is that only ARGs present in the database can be detected; novel ARGs cannot be identified by sequence matching alone. After training with the sequence of the existing datasets, AI can solve the problem and find candidate ARG.

Gustavo et al. developed a deep learning-based tool for predicting ARGs and their classifications from metagenomic sequencing data. The model was trained on ARG sequences sourced from published datasets annotated with antibiotic resistance keywords [10]. DeepARG employs a fully connected neural network architecture with hidden layers containing 2000, 1000, 500, and 100 units, respectively. Feature representation utilizes a dissimilarity-based classification approach. Distances between input DNA sequences and reference sequences are calculated by normalizing DIAMOND-derived bit scores from sequence alignment, where smaller distances indicate higher similarity to known ARGs. The output layer comprises 30 units corresponding to distinct antibiotic resistance categories, employing a softmax activation function to generate categorical probabilities. DeepARG offers two operational modes: DeepARG-SS for short reads (NGS) and DeepARG-LS for long reads (third-generation sequencing). Model validation encompassed predictions of known ARGs, candidate ARGs, an in-silico spike-in experiment, and pseudoARGs. Performance evaluation demonstrated high precision and recall. DeepARG is not only a high-performance tool for ARG prediction but also contributed to the development of a new ARG database, DeepARG-DB.

Antibiotic resistance category prediction is critical, and insights into resistance mechanisms and gene mobility provide a much deeper understanding of identified ARGs. An end-to-end hierarchical multi-task deep learning framework for ARG annotation (HMD-ARG) was designed, operating without alignment to existing sequence databases [11]. The framework comprises a three-level CNN framework for hierarchical ARG annotation. Level 0 determines whether an input sequence is a putative ARG, Level 1 predicts its antibiotic class, resistance mechanism, and mobility status, and Level 2 assigns predicted β-lactamases to specific subclasses (Fig. 5A). Owing to the refinement of ARG databases and the application of CNNs, HMD-ARG demonstrates superior accuracy, precision, and recall compared to DeepARG, with a lower false negative rate. Although AI-based approaches can greatly expand the discovery of putative resistance-associated sequences, computational predictions alone do not prove antimicrobial resistance function. HMD-ARG therefore illustrates how computational predictions can be combined with functional experiments to validate candidate ARGs as previously uncharacterized resistance genes. Eight ARG candidates predicted by HMD-ARG from a clinical *Pseudomonas aeruginosa* strain were expressed in *Escherichia coli*, and antibiotic growth assays provided experimental support for their predicted resistance phenotypes.

The implementation of appropriately complex models can also enhance predictive performance. Recent studies utilized an unsupervised autoencoder architecture to construct a deep neural network ARGNet [12]. An encoder distills salient features through dimensionality reduction, transforming the input data into a compressed latent representation. A decoder reconstructs the original data from this compressed latent space. AI-based tools for ARG detection, from DeepARG to ARGNet, have been developed in recent years. These tools demonstrate enhanced performance, improved multitasking capabilities, and leverage increasingly advanced AI algorithms and architectures [10], [11], [12].

ARGs are frequently disseminated among bacteria through horizontal gene transfer (HGT), facilitated by MGEs. Common types of MGEs include plasmids, transposons, and integrons, which mediate the mobilization of ARGs via mechanisms such as conjugation, transposition, and transformation [13], [14]. It has been widely reported that mobile genetic elements play a critical role in driving the spread of antimicrobial resistance genes. Sequence data serve as a crucial link between MGEs and AMR, providing insights into the mechanisms and origins of antimicrobial resistance [15]. Antonio et al. developed a tool named geNomad, which employs a hybrid approach combining alignment-free and gene-based models to achieve enhanced performance in identifying plasmids and viruses [16]. In the alignment-free component, sequences are one-hot encoded and processed through an IGLOO neural network [17]. The gene-based component utilizes richly annotated datasets containing functional and taxonomic information. The integration of sequence and marker gene data enables the identification of conjugative plasmids carrying AMR genes. A comparison of ARG detection tools is summarized in Table 1. However, Genotype–phenotype concordance varies across ARGs, and the detection of an ARG may not always correspond to phenotypic resistance. This limitation was particularly evident for *P. aeruginosa*, in which detected ARGs showed limited concordance with phenotypic resistance [18]. Accordingly, ARG-based resistance predictions should be linked to the corresponding pathogen, benchmarked against phenotypic AST, and validated in an independent cohort.

**Table 1:** Comparison of AI-based sequence and physical-property models for antimicrobial resistance diagnosis.

Model (year)	Dataset and training size	Algorithm	Performance	Validation	Clinical readiness	Characteristics
Sequence- and mobile-element models						
DeepARG (2018)	14,933 non-redundant ARG sequences from CARD, UniProt, and ARDB; 80:20 training/test split.	Alignment-based feed-forward DNN; DeepARG-SS for short reads and DeepARG-LS for full-length genes.	DeepARG-SS: precision 0.97, recall 0.91; DeepARG-LS: precision 0.99, recall 0.99.	MEGARes benchmark no clinical validation	Research use only; phenotypic confirmation required	Supports both short reads and full-length genes and covers broad ARG classes; performance remains dependent on the reference databases used.
HMD-ARG (2021)	17,282 curated protein sequences from CARD, AMRFinder, ResFinder, ARG-ANNOT, DeepARG, MEGARes, and Resfams	Three-level hierarchical multi-task 1D-CNN on one-hot protein sequences.	ARG detection: accuracy 0.948, precision 0.939, recall 0.971, F1 0.948. Beta-lactamase subclass prediction: Accuracy 0.995, precision 0.989, recall 0.993, and F1 0.991.	Mustard human-gut dataset; eight predicted ARGs were experimentally validated in *E. coli*	Research use only; experimentally supported but not validated for clinical AST.	Alignment-free hierarchical output; protein-only input and fixed-length constraints.
ARGNet (2024)	27,464 curated ARG amino-acid sequences covering 36 categories; viral sequences used as non-ARG controls.	1D convolutional autoencoder for ARG detection; CNN with attention mechanism for class prediction.	Long AA sequences: Precision 0.994, recall 0.960, F1 0.977, and accuracy 0.986 Long nucleotide sequences: Precision 0.989, recall 0.938, F1 0.963, and accuracy 0.976. Short AA sequences: Precision 0.986, recall 0.936, F1 0.960, and accuracy 0.980. Short nucleotide sequences: Precision 0.989, recall 0.919, F1 0.952, and accuracy 0.911.	held-out and quasi-negative test sets, including bacterial non-ARGs and 9903 mcr genes; no clinical validation	Research use only; does not establish phenotypic resistance.	Alignment-free; DNA or protein and short or long sequences; does not capture SNP-only resistance.
geNomad (2023)	∼232 million proteins clustered to build 227,897 marker profiles; 1,000,000 simulated communities used for calibration network.	Hybrid deep-learning and marker-based framework combining IGLOO encoder, XGBoost classifier, attention-based integration, and CRF-based provirus detection.	Plasmids: MCC 77.8%, sensitivity 89.8%, precision 70.8%. Viruses: MCC 95.3%, F1 97.3%	five cluster-separated train/test splits; evaluation on diverse genomic benchmarks; no clinical validation.	Mature research tool; not intended for phenotypic AST.	Detects plasmids, viruses and proviruses; annotates AMR markers but is not an ARG phenotype predictor.

Novel physical property-based technologies						
DhLSI-ANN (2025)	*E. coli* and *E. faecalis* exposed to ampicillin or gentamicin a 10-s holographic video contained 600 frames at 500 × 500 pixels	ANN with 300 input neurons, one 300-neuron ReLU hidden layer, and a two-neuron inhibited/non-inhibited output for pixel-level classification.	phenotypic AST within 2–3 h at 10^3^ CFU/mL; AUROC and precision not reported	comparison with broth microdilution reference labels; wet-laboratory validation; no external laboratory and clinical cohort.	Early-stage; laboratory proof-of-concept only.	Label-free and rapid at low bacterial load; evaluated on only two bacterial species and two antibiotics
Raman-aNN for EVs (2022)	Raman database contained 4335 spectra produced by six bacterial species	CNN-based attentional neural network with channel- and wavenumber-attention modules; integrated-gradient interpretation.	Gram-level classification. Accuracy 96.49 ± 1.56%; Species-level classification. Accuracy 96.02 ± 1.12%; Antibiotic-resistant classification. Accuracy 93.97 ± 2.02%; Physiological-state classification. Accuracy 87.06 ± 2.22%.	wet-laboratory validation; no external laboratory and clinical cohort.	Early-stage; analytical proof-of-concept, not direct AST.	Label-free, multilevel EV classification with interpretable bands; indirect evidence of resistance.
Raman-CNN for *C. auris* (2024)	3652 single-cell spectra from 42 strains across six Candida species;	1D CNNs for Raman spectral classification, with tenfold cross-validation for model selection and spectral feature extraction for identifying resistance- and phenotype-associated peaks	Species/clade identification: accuracy 93.33%, AUROC 0.99; simulated clinical samples: accuracy 98%; fluconazole susceptibility: accuracy 99%; amphotericin B susceptibility: accuracy 94%.	External validation on 900 spectra from nine excluded clinical isolates; clinically derived isolates; no independent external laboratory or multicenter clinical cohort.	Preclinical; supported by laboratory validation.	Combines identification, susceptibility and virulence phenotypes; requires standardization of spectral acquisition and culture conditions
MALDI-TOF–MLP for bovine mastitis *S. aureus* (2021)	82 isolates from 67 cows on 24 farms; repeated train/test evaluations over 30 runs.	Ten supervised machine-learning classifiers, including SVMs, random forest, multilayer perceptron, AdaBoost, logistic regression, LDA/QDA, decision tree, and naive Bayes, evaluated using repeated nested cross-validation; protein-network interpretation.	Benzylpenicillin-resistance classification: Best result from RBF-SVM accuracy 97.54 ± 1.91%, sensitivity 99.93 ± 0.25%, specificity 95.04 ± 3.83%; Multidrug-resistance classification.: Best result from LDA: accuracy 96.81 ± 0.43%, sensitivity 99.88 ± 0.41%, specificity 95.96 ± 0.52%,	isolates originated from 24 farms	Early translational stage; retrospectively evaluated in veterinary samples.	One Health relevance and interpretable protein markers;
MALDI-TOF–ANN for CRKP (2023)	2683 *K. pneumoniae* isolates, including 369 carbapenem-resistant and 2314 carbapenem-susceptible isolates.	ANN using 4500 MALDI-TOF intensity features, with two ReLU dense layers, dropout regularization, and a softmax output for CRKP/CSKP classification.	Sensitivity 0.84, specificity 0.84, F1 0.84, accuracy 0.84，AUROC 0.91	Internal independent holdout validation only; no clinical validation	Early clinical stage; supported by retrospective clinical evaluation	Uses routine MALDI-TOF identification spectra; limited by substantial class imbalance and single-center calibration.

LLMs have become a prominent research focus for processing textual information. Similarly, nucleotide sequences can be treated as a form of text data. Fine-tuned DNABERT has been applied to predicting plasmid and chromosomal origins and identifying horizontal gene transfer by measuring sequence similarity based on concepts of semantic similarity from natural language processing. Finally, an ensemble model incorporating both a large language model and a classical neural network was constructed to predict ARGs and their genomic locations [19].

Beyond its role as a rapid source for AMR detection via ARG analysis, metabolic data consistently offer valuable complementary insights. Leveraging established biochemical screening techniques, the mechanisms underlying antibiotic action have been elucidated and can be effectively modeled using an interpretable “white-box” model. Consequently, the significance of metabolites for AMR diagnosis cannot be overlooked [20]. From the perspective of metabolic activity and DNA mutation, the AMR phenotypes of diverse *Mycobacterium tuberculosis* strains were successfully predicted. This was achieved through the application of Metabolic Allele Classifier (MAC), a novel interpretable AI framework. MAC integrates a metabolic model with a machine learning classifier, thereby revealing functional mechanisms underlying Genome-Wide Association Study (GWAS) data [21].

### 3.2 AI for harnessing signals in novel physical property-based technologies

To address the limitations of traditional AST, which is often time-consuming and labor-intensive, various novel techniques leveraging physical properties and biophotonic technologies have been developed to accelerate the process, even at low bacterial concentrations (Fig. 2). For example, Yang et al. combined a dynamic holographic laser speckle imaging (DhLSI) system with an artificial neural network to enable rapid assessment of bacterial susceptibility [22]. This approach achieves early detection and rapid determination within 2–3 h, even at low concentrations of 10^3^ CFU/mL. The ANN serves as a predictive tool, using input features derived from principal component analysis (PCA) processed data to predict the MIC.

**Fig. 2:**
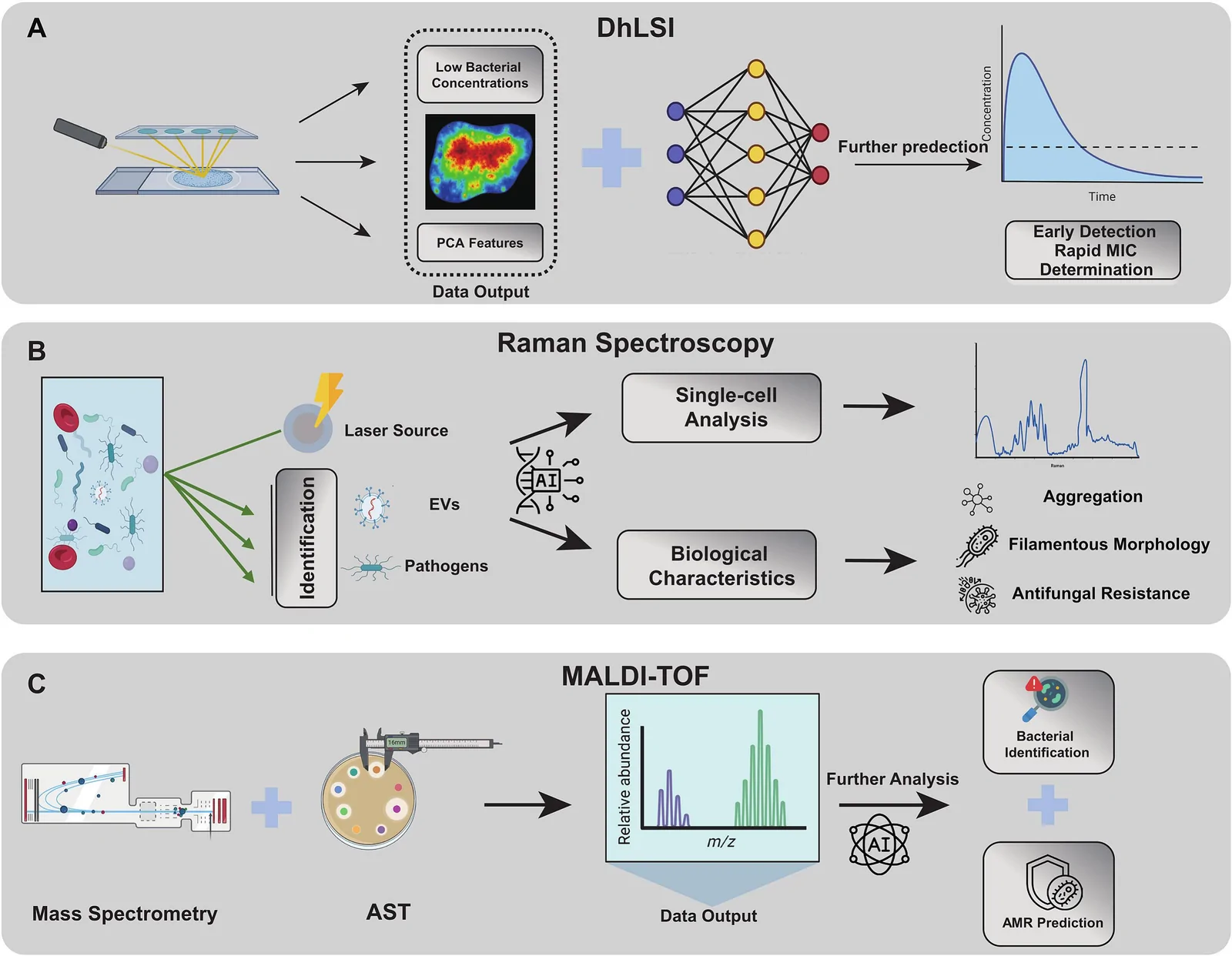
The figure provides an overview of three advanced diagnostic technologies that utilize machine learning and data analysis for the rapid detection and characterization of AMR. (A) DhLSI; (B) Raman spectroscopy; (C) MALDI-TOF. DhLSI, dynamic holographic laser speckle imaging; MALDI-TOF, matrix-assisted laser desorption/ionization time-of-flight.

Raman spectroscopy provides molecular fingerprints through inelastic scattering and can be combined with neural networks for pathogen characterization. A convolutional-attention model characterized pathogenic bacterial extracellular vesicles [23], while a Raman-CNN approach identified single *Candida auris* cells and predicted antifungal resistance, aggregation, and filamentous morphology [24].

AMR-associated changes in protein expression can be detected by combining MALDI-TOF with ML for resistance prediction [25]. Zhang et al. integrated MALDI-TOF mass spectrometry data with AST results and applied an artificial neural network to identify carbapenem-resistant *Klebsiella pneumoniae* and to predict AMR profiles in *K. pneumoniae*, thereby facilitating the selection of more targeted antibiotics [26]. Cecilia et al. utilized MALDI-TOF to predict antibiotic resistance in Gram-negative bloodstream infections using a multivariable logistic regression classifier [27]. Although these studies show rapid advances in methods and algorithms, model evaluation deserves equal attention. A review of clinical risk prediction models likewise reported heterogeneity in model performance, limited external validation, and insufficient assessment of calibration and clinical utility [28]. AMR models should therefore be tested in external populations, checked for well-calibrated predictions, and evaluated according to their intended clinical role.

## 4 AI-enhanced therapeutic development

Since the discovery of penicillin by Alexander Fleming in 1928, antibiotics have served as the primary therapeutic agents against infectious diseases. However, the use of antibiotics can lead to AMR through mechanisms such as prevention of drug uptake, efflux pumps, target site modification, drug inactivation, and the development of alternative metabolic pathways [29]. These mechanisms render infections more difficult to treat and increase the risk of severe disease and mortality. Moreover, certain pathogens exhibit inherent resistance to existing antibiotics. Therefore, the discovery of novel antibiotics or alternative treatments represents an urgent imperative.

Emerging therapeutic strategies, such as antimicrobial peptides and phage therapy, are already being applied in clinical settings. Nevertheless, the number of available antibiotic agents, whether antibiotics or novel therapeutics, remains limited, and there is a considerable need for more efficient antimicrobial agents. Although vast libraries of small molecules, antimicrobial peptides (AMPs), and phages exist, a major challenge lies in the identification of candidates with potent antimicrobial activity. Advances in artificial intelligence, with its powerful learning capacity and big-data processing capabilities, offer promising avenues to accelerate the discovery of natural antimicrobial compounds and even to generate novel agents with enhanced efficacy. Yet, computational generation marks only the entry point to therapeutic translation. As summarized in Fig. 1B, AI-enabled candidates must progress through synthesis and in vitro testing, in vivo evaluation of efficacy and safety, pharmacological and toxicological characterization, and ultimately clinical assessment. Together, these stages bridge candidate discovery and clinical use while maintaining a clear distinction between preclinical promise and demonstrated therapeutic value. Table 2 summarizes representative AI models used in therapeutic development.

**Table 2:** Performance and translational evidence of representative AI models in therapeutic development and AMR decision support.

Model / study	Core algorithm	Performance metrics	Translational framework
AI-enhanced therapeutic development			
SyntheMol (2024)	Reaction-constrained MCTS guided by Chemprop-based and random-forest antibacterial activity predictors.	Experimental hit rate: 6/58 (10.3%) synthesized candidates showed antibacterial activity and structural novelty.	**Computational validation:** searched a nearly 30-billion-molecule synthesizable space using predicted antibacterial and toxicity objectives **In vitro:** 58 molecules synthesized; six actives against *A. baumannii*, with some broader antibacterial activity
De novo antibiotic design pipeline (2025)	Chemprop D-MPNN scoring plus CReM genetic search, F-VAE and JT-VAE generation	Experimental hit rate: 7/24 (29.2%) synthesized candidates showed selective antibacterial activity. Lead activity: NG1 and DN1 were active against multidrug-resistant isolates and efficacious in mouse infection models.	**Computational validation:** >45 million fragments screened and > 36 million compounds generated across fragment-based and de novo routes **In vitro:** NG1 and DN1 were active against multidrug-resistant isolates and were investigated mechanistically **In vivo:** efficacy in mouse *N. gonorrhoeae* vaginal-infection and MRSA skin-wound models **Pharmacological and toxicological evaluation:** cell cytotoxicity, human-RBC haemolysis, modified Ames genotoxicity and acute mouse tolerability
Human-gut AMP mining pipeline (2022)	Ensemble of LSTM, attention and BERT sequence classifiers	Experimental hit rate: 181/216 (83.8%); 10/11 top candidates were active against clinical multidrug-resistant isolates. In vivo: three leads reduced mouse lung bacterial burden by >10-fold.	**Computational validation:** trained on four public AMP databases and applied to 11,011 metagenomes; 2349 candidates prioritized **In vitro:** 216 peptides synthesized; 181 active and 10/11 top peptides active against clinically isolated multidrug-resistant bacteria **In vivo:** three leads reduced bacterial burden in a *K. pneumoniae* mouse lung-infection model **Pharmacological and toxicological evaluation:** haemolysis and mammalian-cell cytotoxicity
AMP-GPT (2025)	GPT-2 peptide language model with contrastive prompt tuning, distillation and reinforcement learning	In silico activity: 83.4% of generated peptides were classified as active by all three external predictors. Wet-lab validation: 18 peptides were synthesized, and two lead candidates were identified; 3/5 *P. acnes* candidates showed high potency.	**Computational validation:** pretrained on 630,683 UniProt peptides; prompt tuning and MIC-guided reinforcement learning; **In vitro:** KW13 and AI18 showed broad activity against resistant clinical Gram-negative isolates; **In vivo:** therapeutic efficacy in a bacterial-pneumonia mouse model **Pharmacological and toxicological evaluation:** RBC haemolysis and plasma-stability testing; both leads had low haemolysis and favorable stability
Latent-diffusion AMP generator (2025)	Latent diffusion in a VAE-learned peptide latent space for AMP generation	Experimental hit rate: 25/40 (62.5%); nine peptides had MIC <25 μM. Safety: AMP-24 caused <5% haemolysis at 200 μM, with a safety margin ≥32× MIC.	**Computational validation:** conditional generation and in-silico prioritization of diverse antimicrobial peptide sequences **In vitro:** 40 peptides synthesized; 25 actives; AMP-24 and AMP-29 selected for mechanistic studies **Pharmacological and toxicological evaluation:** rat-RBC haemolysis and HaCaT/HUVEC cytotoxicity; AMP-24 caused <5% haemolysis at 200 μM and had a safety margin ≥32-fold over MIC
EvoGradient (2025)	Explainable AMP potency networks plus projected gradient-based virtual sequence evolution	Wet-lab validation: all six top evolved peptides selected for synthesis were active against multidrug-resistant pathogens. In vivo: pep-19-mod reduced bacterial load by >95% in the mouse thigh-infection model.	**Computational validation:** 32 peptides from low-abundance oral bacteria were virtually evolved for higher predicted potency **In vitro:** six top candidates synthesized and active against carbapenem-resistant Gram-negative bacteria and vancomycin-resistant *E. faecium* **In vivo:** pep-19-mod tested by systemic and local administration in a mouse thigh-infection model
deepAMP (2024)	Transformer peptide language model with masked-sequence generation and iterative activity optimization	Designed candidates: >90% of 29 outperformed penetratin; T2–9 showed 10.6–64-fold lower MICs across five bacteria. In vivo: three leads reduced an approximately 10^7^-CFU mouse wound burden to near zero at 24 h.	**Computational validation:** pretrained on 300,000 peptides and fine-tuned on paired degradation/optimization data **In vitro:** 29 candidates tested; three leads showed broad activity, biofilm inhibition and no resistance after 25 *S. aureus* passages **In vivo:** three leads reduced ∼10^7^ CFU to approximately zero at 24 h in a *P. aeruginosa* mouse-wound model **Pharmacological and toxicological evaluation:** RBC haemolysis, mammalian-cell cytotoxicity, mouse blood testing and organ histology;
TransSAFP (2025)	Pretrained peptide encoder with transfer learning, noise augmentation and sample weighting	Experimental hit rate: 121/140 (86.4%) at MIC ≤100 μg/mL. Lead p45: broad activity, biofilm eradication and efficacy in a mouse intestinal-infection model	**Computational validation:** few-shot prediction of functional self-assembling peptides containing natural and non-natural amino acids **In vitro:** 140 candidates synthesized; lead p45 showed broad activity, biofilm eradication and no acquired resistance **In vivo:** p45 showed efficacy in a mouse intestinal bacterial-infection model **Pharmacological and toxicological evaluation:** in-vivo toxicity testing of the lead and in-vitro selectivity and safety assays were performed
PhageTB (2023)	Ensemble of alignment-based scores and alignment-free machine-learning classifiers	Independent-validation accuracy: 67.9%, 80.6%, 85.5%, 90.0% and 93.5% at genus, family, order, class and phylum levels, respectively.	**Computational validation:** 826 phage–host interactions used for development **External validation:** independent 1201-interaction dataset; geographic and temporal structure were not reported
PhageHostLearn (Boeckaerts et al., 2024)	ESM-2 multi-instance embeddings + XGBoost for pairwise phage–host prediction.	Discrimination: LOGOCV AUROC up to 0.818; independent in-vitro AUROC 0.793. Prospective testing: top-five hit ratio 93.8% among isolates with a confirmed interaction	**Computational validation:** leave-one-group-out cross-validation grouped by K-locus identity **External validation:** independent prospective isolate set, 28 high-risk Spanish clinical isolates; **In vitro:** top five phages per isolate tested by replicated spot assays
Predictive phage-therapy model (Keith et al., 2024)	Random forests	Generalist-phage models: F1 > 0.60. Predicted bespoke cocktails outperformed the general cocktail in vitro.	**Computational validation:** 31 phage models developed from activity measurements against 314 *E. coli* urinary isolates **External validation:** 10 newly isolated clinical UPEC strains not used in training **In vitro:** predicted and observed bespoke cocktails compared in artificial and canine urine; an additional general cocktail was validated on 50 isolates
NDM-inhibitor XGBoost ensemble	Ensemble of 18 XGBoost classifiers using molecular descriptors	Discrimination: AUROC 0.8095 in the training set and 0.7582 in the scaffold-split test set. Prospective quantitative high-throughput screening(qHTS): 164/2816 (5.82%) ML-selected compounds were classified as active versus 118/9856 (1.19%) in the comparator set.	**Computational validation:** scaffold split: 33,032 training and 10,395 test compounds **External validation:** chemical-space rather than geographic/temporal validation: independent NCATS Genesis library of 76,369 compounds, with 2816 prioritized for qHTS **In vitro:** biochemical NDM-1 inhibition and bacterial assays; selected hits restored meropenem activity against resistant *E. coli* **Pharmacological and toxicological evaluation:** concentration–response IC50 and antibacterial combination-response profiling;

AI for AMR combat and accessible question-answering			
SARPLLM (2025)	Qwen2 with LoRA fine-tuning after feature selection	3-fold cross-validation with four repeated tests (50 selected genomic features): mean F1 0.938 (AUG), 0.985 (AXO), and 0.913 (CHL).	**Computational validation:** 1167 US NARMS/NCBI *S. typhimurium* genomes; repeated cross-validation only
EUCAST-GPT-expert	Customized GPT-4 agent grounded in EUCAST guidance and breakpoint rules	ESBL: sensitivity 95.4%, specificity 69.2%. AmpC: sensitivity 96.9%, specificity 86.3%. Carbapenemase: sensitivity 100%, specificity 98.8%. Three-run concordance: 81.9%.	**Computational validation:** three repeated prompts compared with routine diagnostics, three microbiologists and uncustomized GPT-4 **Clinical evaluation:** retrospective single-laboratory diagnostic benchmark using 225 Gram-negative clinical isolates and 862 phenotype categories; no prospective workflow or patient-outcome evaluation
Fourteen-LLM antibiotic benchmark (2025)	General-purpose conversational LLMs evaluated without task-specific retraining	ChatGPT-o1: appropriate drug selection 71.7%, appropriate dosage 96.7%. Gemini: appropriate overall recommendations 75.0%	**Computational validation:** blinded expert review of 14 chatbots on 60 curated clinical vignettes spanning 10 infection types
Fine-tuned GPT-3.5 literature miner (2025)	Fine-tuned GPT-3.5-turbo	Held-out test (23 papers): nine categorical extraction fields averaged >80% accuracy versus 62.7% for the pretrained model.	**Computational validation:** 52 papers used for training and 23 for testing within a 75-paper curated corpus; 1012 peptide-assembly entries
ARG dissemination random forest (Lund et al., 2025)	Random forest using genetic compatibility, ecological co-occurrence and ARG features	General model: mean AUROC 0.873, sensitivity 0.806, specificity 0.785. Mechanism-specific models: AUROC 0.821–0.926.	**Computational validation:** cross-validation and permutation analyses across ∼1 million genomes and > 20,000 metagenomes; globally pooled data were not a geographic external holdout
Poultry One-Health models (Baker et al., 2023)	Randomized trees using metagenomic species and ARG abundances	Nested CV: 10/17 antibiotic-specific models achieved AUROC >0.90.	**Computational validation:** 461 microbiomes from 10 farms and four abattoirs in three Chinese provinces over 2.5 years;
Priority-drug spatial ensemble (Zhao et al., 2024)	Gaussian process stacked generalization combining boosted regression trees, LASSO logistic regression, and a feed-forward neural network.	Spatial CV: AUROC 0.880–0.994 for predicting resistance prevalence >50%,	**Computational validation:** spatial cross-validation on 1088 point-prevalence surveys from LMICs, 2000–2019;
AMR-Policy GPT (2025)	RAG with BGE-M3 embedding-based retrieval, reranking, and GPT-4 generation.	87.3% accuracy in financial-plan extraction and 93.1% efficacy for multilingual queries.	**Computational validation:** prototype question-answering and policy-synthesis tests over 383 multilingual AMR policy documents from 146 countries; manual checking reported

### 4.1 Discovery of antimicrobial candidates

The advent of AlphaFold3 and the maturation of chemical libraries have significantly advanced AI-driven drug design. Currently, the predominant AI approach for discovering novel antibiotics involves screening compounds from existing chemical libraries. However, this method is time-consuming and inherently limited, as it cannot identify candidate molecules absent from the library, which itself represents only a fraction of the total chemical space. Consequently, generative AI for de novo antibiotic design represents an emerging research focus, with the molecular representation method maturing (Fig. 3).

**Fig. 3:**
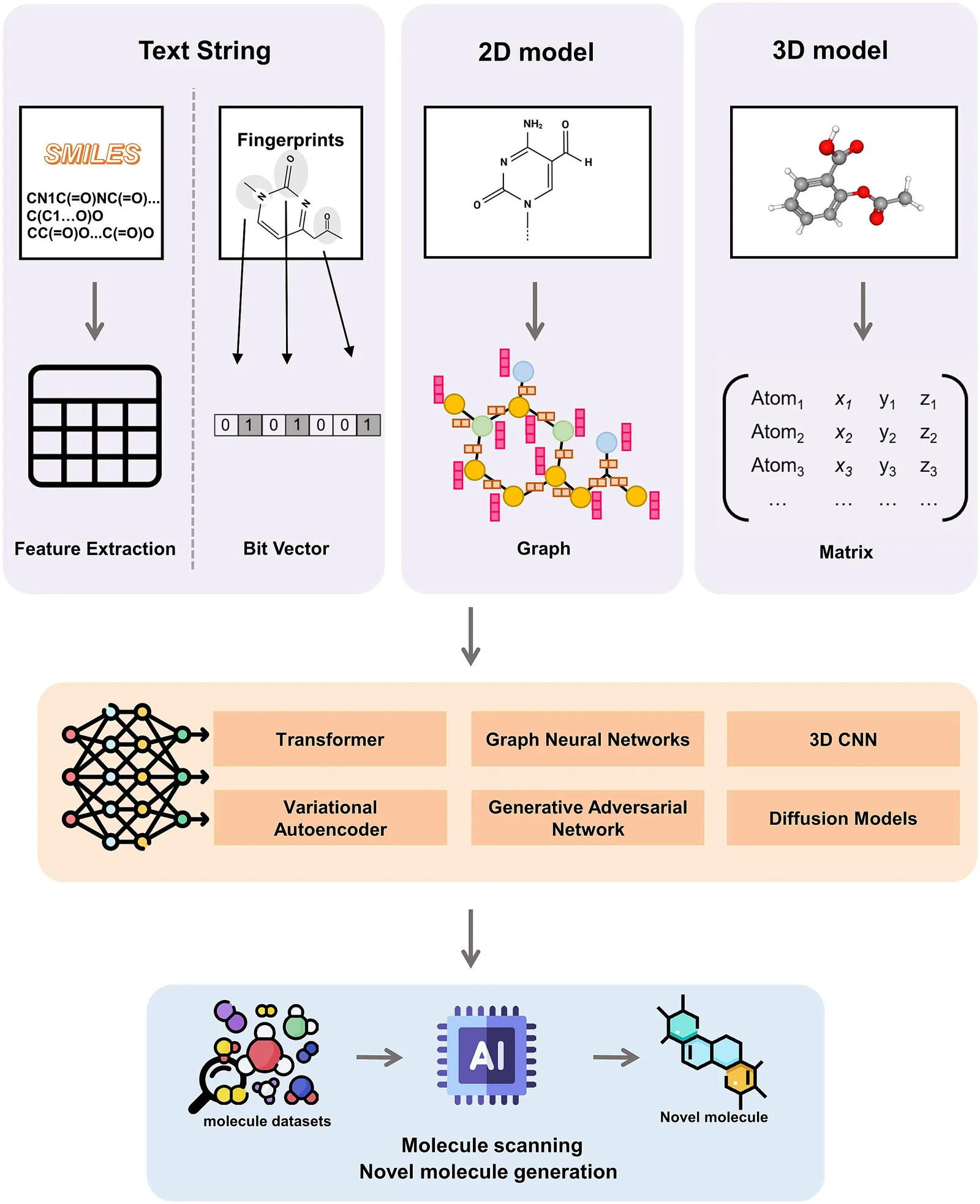
The figure illustrates the process of molecule representation for AI. It shows how a chemical structure (top) is translated into different data formats that can be processed by AI. Text String Representation: Molecules are encoded as text. SMILES use a string of characters to describe the molecule's connectivity. Molecular fingerprints represent the presence or absence of specific substructures as a binary vector. 2D Model Representation: A molecule is treated as a graph, where atoms are nodes and bonds are edges. This representation captures the topological structure of the molecule. 3D Model Representation: This method captures the molecule's three-dimensional spatial arrangement. The coordinates of each atom (x, y, z) are stored in a matrix, providing a more detailed view of the molecule's shape and conformation. These representations can serve as inputs to AI models such as Transformers, graph neural networks, 3D CNNs, variational autoencoders, generative adversarial networks, and diffusion models. SMILES, Simplified Molecular Input Line Entry System; CNN, convolutional neural network.

Chemprop serves as a foundational tool for predicting chemical properties, utilizing a state-of-the-art method known as Directed Message Passing Neural Networks (D-MPNNs) [30]. This approach represents molecules as graphs, where atom and bond information is incorporated into the embedding. Chemprop has become an important tool for evaluating the potential efficacy of molecules generated by AI methods for antibiotics. Swanson et al. constructed an easily synthesizable chemical space by linking two or three molecular building blocks (totaling 132,000) using 13 predefined chemical reactions [31]. Subsequently, a generative model named SyntheMol employed Monte Carlo tree search to explore this generated molecular space, guided by properties predicted by Chemprop. Of 58 synthesized molecules, six showed measurable antibacterial activity against *Acinetobacter baumannii* in vitro. This study therefore demonstrated both synthetic feasibility and experimental antibacterial activity. However, these compounds remain at the preclinical stage, as further pharmacological characterization and clinical evaluation are still required before clinical translation.

Another study also developed a generative deep learning approach for de novo antibiotic design utilizing a fragment-based method. Chemprop was employed to predict and filter fragments possessing antibacterial properties. The study made use of two machine learning methods for de novo compound design: one leveraging a CReM-based genetic algorithm and the other a fragment-based variational autoencoder (F-VAE) [32]. These algorithms generated novel antibiotics using the selected antibacterial fragments as input. Two lead compounds, NG1 and DN1, were further evaluated for cytotoxicity, haemolysis, in vivo efficacy, mechanisms and activity against multidrug-resistant isolates. This study provides a strong example of integrating computational generation with comprehensive pharmacological evaluation (Fig. 5B).

### 4.2 Discovery and design of candidate AMP for antimicrobial treatment

AMPs, originally derived from natural sources, exhibit broad-spectrum antimicrobial activity with high specificity and low toxicity. Notably, they demonstrate a lower propensity for inducing AMR and stronger phylogenetic barriers within bacteria against horizontal transfer of developed resistance compared to conventional antibiotics [15]. Their antimicrobial effects commonly involve membrane destabilization or transmembrane pore formation. Because AMPs are generally only 10–60 amino acids long, conventional alignment-based methods have limited sensitivity for their discovery [33]. AI-assisted workflows now support AMP mining, de novo generation, sequence optimization, and integration with functional materials.

Large-scale metagenomic studies demonstrate the scalability of AI-based AMP mining. Ma et al. developed a computational pipeline for candidate AMP screening using human gut microbiome data, incorporating natural language processing models such as LSTM [34], Attention [35], and BERT [36], [37]. The researchers trained an AMP prediction model based on four public databases: ADAM, APD, CAMP, and LAMP. For AMP mining, the prediction model was applied to 11,011 metagenomes, leading to the identification of 2349 candidate AMPs. Among 216 peptides chemically synthesized, over 83% exhibited high antimicrobial activity, as confirmed by experimental validation. Santos-Júnior et al. subsequently used Macrel to screen 63,410 metagenomes from diverse habitats [38]. This analysis generated AMPSphere, which contains 863,498 non-redundant peptides, most of which were absent from existing databases [39].

Protein language models (PLM), analogous to natural language models, are capable of processing amino acid sequences as if they were sentences, leveraging long-range contextual information to achieve a deeper understanding and feature extraction from protein sequences with the concept of evolution [40]. ESM-2 represents the current state-of-the-art PLM, which transforms protein sequences into feature vectors encoding structural, functional, and evolutionary information. These embeddings facilitate improved computational interpretation and analysis. By simplifying the neural architecture and eliminating the requirement for multiple sequence alignment (MSA), ESMFold [41] achieves superior inference speed compared to AlphaFold [42] and RoseTTAFold [43]. Owing to its high efficiency and relatively simple architecture, ESM-2 has been widely adopted in research addressing AMPs.

A species-aware, peptide-tuned ESM-2 model was developed for AMP identification through pre-training on 1.7 million peptides. During fine-tuning, the embeddings generated by the peptide-tuned ESM-2 were integrated with genomic features from 45 different species to learn species-specific peptide activities and MIC values [44]. This species-aware approach enhances the efficiency of AMP design and screening processes tailored to particular species.

In addition to sequence-derived information, incorporating features such as three-dimensional structure, surface geometry, and physicochemical properties can lead to a more comprehensive model. Sun et al. embedded peptide sequences using both ProteinBert [45] and ESM-2 and predicted 3D structures with AlphaFold2. Protein structural and physicochemical features were represented using graph- and surface-based approaches and integrated through neural networks for AMP classification [46], [47].

AMP mining involves searching for candidate molecules within the vast peptide sequence space, which comprises approximately 4.5 × 10^41^ possibilities [48]. As an alternative approach, AI has been employed to de novo design AMPs with desired properties.

For instance, Wang et al. developed a novel AMP design strategy using an LLM-based foundation model. They trained AMP-GPT on peptide sequences sourced from UniProt and integrated techniques such as prompt tuning, contrastive learning, knowledge distillation, and reinforcement learning [48]. This approach enabled rapid de novo generation of highly diverse and potent AMPs tailored to target bacterial species. Several studies have further developed end-to-end AI frameworks that cover the entire process of AMP design and activity prediction, such as MIC, leveraging methods based on LLMs or ESM-2 [49], [50].

Whether through AMP mining or de novo AMP design, initially identified antimicrobial peptides may not fully meet clinical requirements. Therefore, peptide optimization plays a crucial role in enhancing their therapeutic potential. Wang et al. developed a virtual evolution model named EvoGradient, which evolves candidate AMP sequences through an iterative process combining gradient descent with projection to identify locally optimal sequences near the original peptide [51]. Another study used optimization models to improve peptide antimicrobial activity and membrane permeability, resulting in broader-spectrum activity, stronger membrane disruption, reduced cytotoxicity, and increased sequence diversity [52], [53].

The integration of AMPs with bioinspired materials represents another promising strategy to enhance the efficacy and functionality of AMP applications. By introducing self-assembling moieties into AMPs, self-assembling functional peptides (SAFPs) can be created de novo. The antimicrobial activity of these SAFPs was evaluated by measuring MIC, which served as input for model training [54]. Initial pretraining was conducted using a public dataset of peptides composed of the 20 natural amino acids, followed by transfer learning with SAFP data. This approach enables high-throughput prediction of SAFPs with potential antimicrobial activity.

Collectively, these studies demonstrate different levels of experimental maturity. Although some studies remain limited to computational screening, peptide synthesis, and in vitro activity testing, Ma et al. and subsequent AI-guided frameworks have extended validation to include in vivo safety assessment and efficacy testing in mouse infection models [36], [48], [50]. Nevertheless, most AI-designed AMPs remain early-stage candidates because comprehensive pharmacological evaluation, including PK/PD characterization, immunogenicity, long-term toxicity, off-target effects, resistance emergence, and clinical studies, remains incomplete.

### 4.3 Bacteriophage treatment

Phage therapy has emerged as a promising alternative to antibiotics. Phages are naturally occurring, highly specific, nontoxic, and effective against antibiotic-resistant bacteria, offering a potential solution to the challenge of AMR [55]. However, phage selection remains challenging because of extensive phage diversity and highly strain-specific, often poorly characterized host interactions. Inappropriate phage selection may also promote the spread of AMR or virulence genes. AI can facilitate phage–host prediction and support safer phage selection and design.

Understanding host–phage interactions is fundamental to developing phage-based therapies. Aggarwal et al. developed PhageTB to address three key tasks: 1) predicting the bacterial strain from a phage genome sequence; 2) predicting host–phage interactions; and 3) identifying the most suitable phage to lyse a given bacterial strain [56]. PhageTB employs an ensemble model that integrates AI methods with alignment-based strategies to accomplish these tasks. Wei et al. predicted host–phage interactions by using mutual information to enhance phage features, combined with regional graph convolution, which learned meaningful representations from densely connected, sequence-based heterogeneous graphs, and incorporated a regional-level attention mechanism to quantify the contribution of neighbors from different regions to the central node [57].

From predicting simple host-phage interactions to selecting therapeutic phages, the application of phage therapy has moved closer to clinical implementation. Receptors and phage receptor-binding proteins (RBPs) play a pivotal role in the efficacy of phage treatment [58]. Boeckaerts et al. used phage receptor-binding proteins and *Klebsiella capsular* polysaccharides as input features, with ESM-2 and XGBoost used to predict phage–host specificity and prioritize candidate phages [59]. Similarly, Keith et al. developed a machine learning approach to predict phage activity, which holds potential for guiding the selection of bespoke phage cocktails [55].

With the advent of genomic language models such as Evo 1 and Evo 2 [60], the de novo generation of novel bacteriophages for therapeutic use has become feasible. Using Evo 1 as a foundation model and ΦX174—which has been completely sequenced and synthesized—as a template, 16 functional phage genomes were designed to target *E. coli* strains, including resistant variants [61]. The first synthetic phage exemplifies a pioneering approach to combating AMR through designed genomes, demonstrating a promising methodology from molecular to genomic scales.

AI-based host–phage prediction addresses only one component of therapeutic phage selection. Candidate phages should be validated experimentally for host range and lytic activity against clinically relevant isolates. Their genomes should be screened for lysogeny-associated genes, toxins, and antimicrobial resistance genes, while clinical translation requires consideration of phage–bacterial evolutionary dynamics, including resistance development, immune neutralization, and PK/PD properties [62]. Robust manufacturing and quality-control procedures are also essential to ensure product quality and safety in accordance with regulatory requirements.

### 4.4 Screening for small molecule enzyme inhibitors

To evade the bactericidal effects of antibiotics, bacteria can produce hydrolytic enzymes that inactivate antibiotic function, thereby contributing to AMR [63], [64]. Consequently, combination therapies utilizing both antibiotics and enzyme inhibitors represent a viable strategy to address this challenge and are currently employed in clinical settings. For example, ceftazidime–avibactam is a combination drug where avibactam inhibits the activity of β-lactamases [65].

However, more potent enzyme inhibitors are needed to assist antibiotics in overcoming AMR. AI and computational technologies have accelerated the discovery of such inhibitors by enabling the screening of molecules against target enzymes. This is achieved through the evaluation of molecular docking scores or the prediction of docking efficiency. For instance, novel BfmR inhibitors, which prevent the inactivation of carbapenems for treating *Acinetobacter baumannii* infections, were identified via virtual screening of compound libraries using a structure-based docking pipeline. This virtual screening relied on the precise structure of the target enzyme and detailed molecular docking simulations of compound-enzyme interactions [66]. Alternatively, an NDM inhibitor was identified using a descriptor-based ML approach rather than molecular docking. Molecular structures were converted into AlvaDesc descriptors and used to train an XGBoost model on high-throughput screening data, with SMOTE applied to address class imbalance between active and inactive compounds. [67].

Compared with the discovery of novel antibiotics and AMPs, enzyme-inhibitor development requires prior identification of a suitable target, followed by inhibitor discovery or generation. Integrated AI pipelines are increasingly combining these steps. For instance, Kamya et al. constructed a comprehensive AI-based pipeline utilizing PandaOmics for target identification and Chemistry42 for inhibitor generation [68]. Using this pipeline, more than 15 inhibitors have been generated for treating human diseases. Examples include a TNIK inhibitor for fibrosis and CDK12/13 inhibitors for cancer treatment [69]. Although Lu et al. focused on inhibitors for human targets, the underlying concept and methodology can be adapted for developing novel enzyme inhibitors targeting bacterial systems. This approach has the potential to accelerate progress in combating AMR.

## 5 AI for AMR combat: leveraging accessible question-answering to support decision-making

The advent of ChatGPT has spurred growing interest in AI language foundation models, which can understand contextual sequences and solve downstream tasks through specialized fine-tuning. LLMs like ChatGPT, Qwen, Claude, and DeepSeek have demonstrated remarkable linguistic and reasoning abilities. Fine-tuned AI agents based on these models have been evaluated for their ability to answer clinical questions with high levels of accuracy and professional relevance. Such systems have already been integrated into clinical systems in some hospitals to assist physicians in clinical decision-making. In one representative study, 92% of the generated answers aligned with established scientific and clinical consensus [70]. Given that sequential medical data and clinical narratives of infected patients can also be interpreted as a form of language, there is a clear advantage in fine-tuning LLMs to improve their comprehension and processing of such specialized information. Consequently, numerous studies are exploring how to equip conversational LLMs with knowledge of AMR to assist in clinical application and decision-making (Fig. 4). Table 2 summarizes representative AI models used in AMR decision support.

**Fig. 4:**
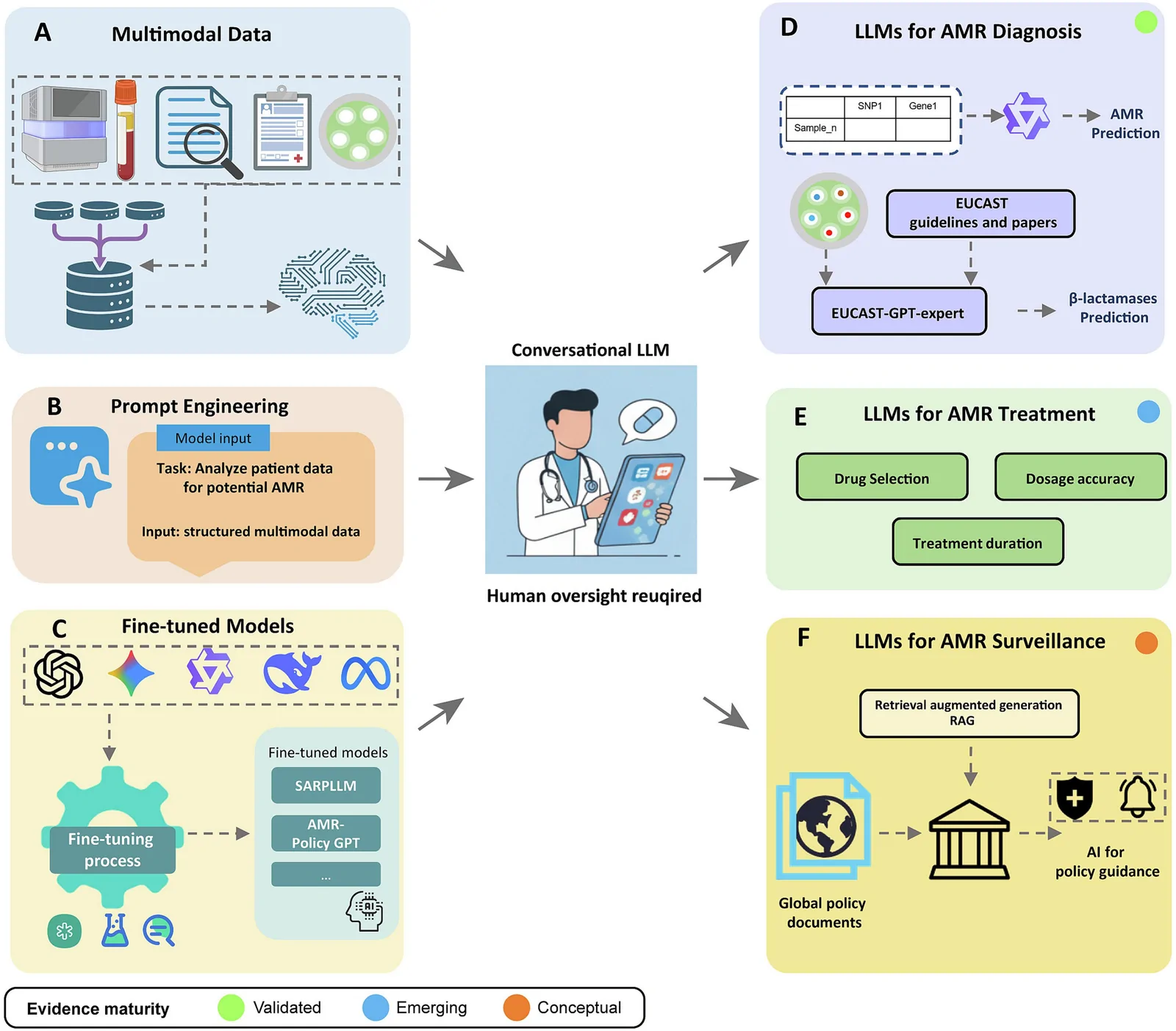
The figure illustrates a conversational LLM framework designed for decision-making. The workflow begins with multimodal data (A), which is collected from various sources like electronic medical records, sequence data, medical images, lab results and clinical guidelines. This data is then used as input for prompt engineering (B), where a specific task is defined for the model. The data is processed by fine-tuned models (C), which are optimized for specific AMR tasks to enhance performance. The system then utilizes LLMs for various functions: LLMs for AMR Diagnosis (D): The system can predict AMR and β-lactamases by integrating data from sources like EUCAST guidelines and academic papers, as well as patient-specific genetic information. LLMs for AMR Treatment (E): The model assists with treatment decisions, including drug selection, dosage accuracy, and determining the duration of treatment. LLMs for AMR Surveillance (F): The system leverages RAG to analyze global policy documents and provide AI-driven guidance for AMR policy and surveillance. The arrows indicate potential functional links rather than a fully implemented or clinically validated workflow. RAG, Retrieval-Augmented Generation.

### 5.1 The application of conversational LLMs in AMR diagnosis

Conversational LLMs have proven useful in assisting clinicians with the diagnosis of AMR by allowing interactive inquiry into a patient's current clinical presentation. Fine-tuning Qwen2 was proved to have the ability to predict *Salmonella* antimicrobial resistance with quantum computing techniques [71]. Qwen2 is a pre-trained LLM that can be adapted to specific tasks using domain-specific data. For *Salmonella* AMR prediction, accessory gene and core SNP matrices obtained from pan-genome analysis were first reduced using chi-square testing and conditional mutual information. Through prompt engineering, the selected features were converted into text-like inputs containing antimicrobial, SNP, and gene information. Qwen2 was then fine-tuned with LoRA to generate SARPLLM, which achieved mean F1 scores of 0.938, 0.985, and 0.913 for AUG, AXO, and CHL, respectively.

In addition to sequence data, which can be processed by LLMs, certain LLMs are also capable of learning from multimodal data that integrate both images and text. AST based on disk diffusion imaging and guidelines for the detection of resistance mechanisms, together with relevant papers and established guidelines, constitute high-quality learning corpora for LLM training and fine-tuning. Giske et al. customized a GPT agent based on GPT-4 to perform pre-classification of disk diffusion results for indicating β-lactamases [72]. The model was first supplied with clinical documents—specifically, the EUCAST guidelines on the detection of resistance mechanisms and specific resistances of clinical and epidemiological importance, along with EUCAST expert rules—enabling the GPT-agent to gain a robust understanding of EUCAST recommendations. Subsequently, the EUCAST-GPT expert was customized using disk diffusion data combined with inhibition zone measurements. The AI agent demonstrated a median sensitivity of 95.4% for ESBL detection, 96.9% for AmpC detection, and 100% for carbapenemase detection, accompanied by a median reasoning length of 158 words (IQR 140–174) (Fig. 5C). Although the performance stability of the EUCAST-GPT expert was comparable to that of human experts, its lower specificity indicated a tendency toward over-flagging. This study demonstrated promising diagnostic performance in AMR diagnosis and underscores its potential for supporting clinical decision-making in this domain, while further evaluation of clinical errors, calibration, reproducibility, clinician interaction, and patient outcomes is needed to establish clinical safety.

**Fig. 5:**
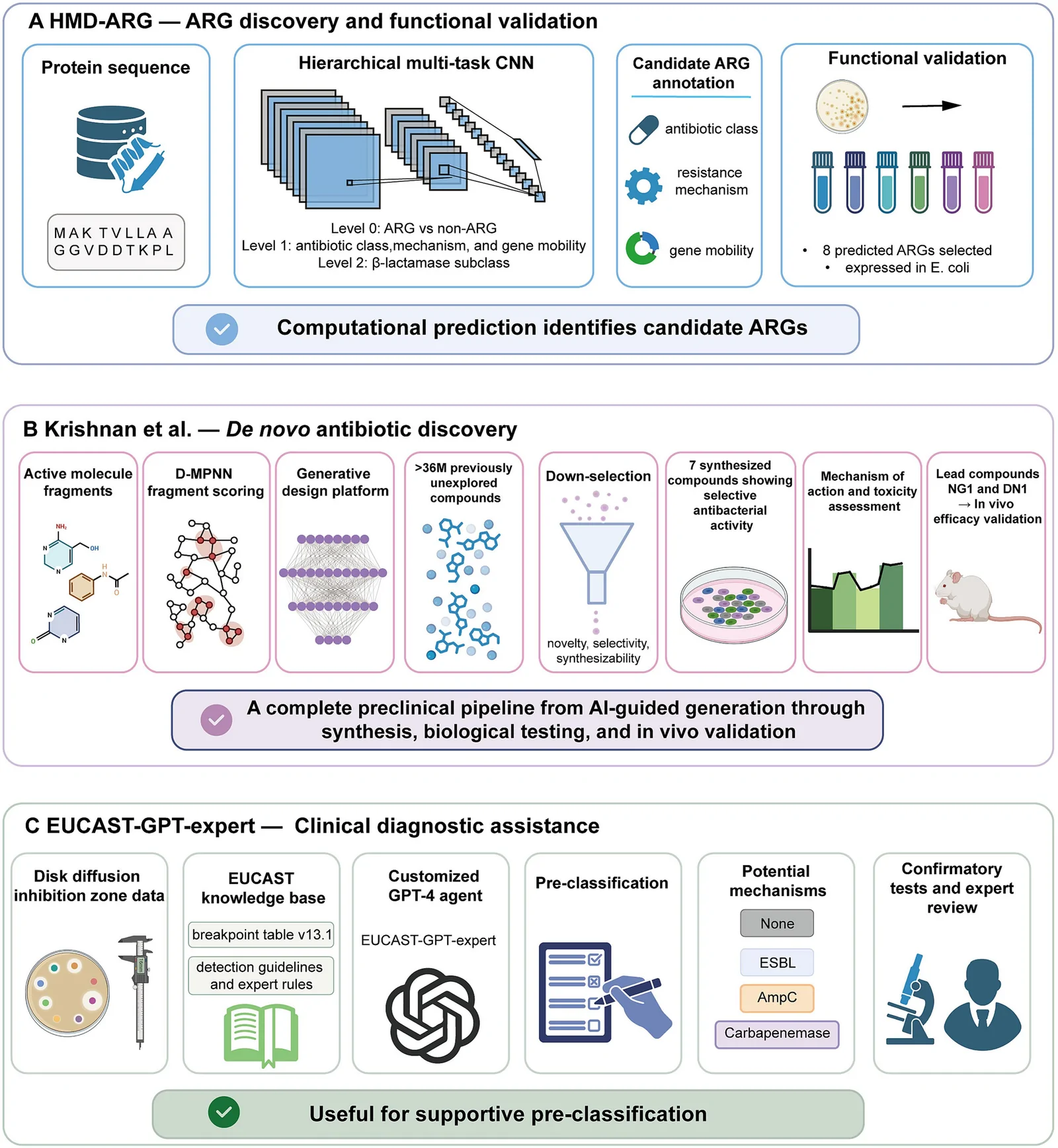
The figure illustrates representative AI applications that connect computational predictions with experimental validation or expert assessment across AMR research. (A) HMD-ARG uses a hierarchical multi-task CNN to identify ARGs from protein sequences and predict antibiotic class, resistance mechanism, gene mobility, and β-lactamase subclass. Eight predicted genes from *Pseudomonas aeruginosa* were expressed in *Escherichia coli*, and antibiotic-exposure experiments supported their resistance functions. (B) Krishnan et al. combined D-MPNN scoring with generative models to generate more than 36 million previously unenumerated compounds. Of 24 synthesized candidates, seven showed selective antibacterial activity. Lead compounds NG1 and DN1 underwent mechanistic, toxicity, and in vivo evaluation in *Neisseria gonorrhoeae* and *Staphylococcus aureus* infection models. (C) EUCAST-GPT-expert integrates disk-diffusion data with a customized GPT-4 agent informed by EUCAST guidance and breakpoint table v13.1. It assigns preliminary categories of none, ESBL, AmpC, or carbapenemase before confirmatory testing and expert review. D-MPNN, directed message-passing neural network; EUCAST, European Committee on Antimicrobial Susceptibility Testing;

### 5.2 The application of conversational LLMs in AMR treatment and literature mining

Conversational LLMs have demonstrated strong capabilities in responding to clinical questions. Within the specific domain of AMR treatment, they also show considerable potential. To evaluate the performance of conversational LLMs in recommending antibiotics across diverse clinical scenarios, De Vito et al. conducted a benchmark study of 14 LLM-based chatbots, including ChatGPT, Claude, Copilot, Gemini, Le Chat, Grok, Perplexity, and Pi.ai. These models were assessed on criteria such as drug selection, dosage accuracy, and treatment duration. The responses generated by each LLM were reviewed by clinical experts to evaluate the appropriateness and accuracy of the recommendations [73]. ChatGPT-O1 exhibited the best performance in both drug selection (correct rate of 71.7%) and dosage (correct rate of 96.7%), while Gemini performed best in providing the most appropriate overall recommendations (correct rate of 75%). Nevertheless, these findings also imply that 25–28% of recommendations may be incorrect. In antimicrobial therapy, such errors could have important clinical consequences, for example by suggesting an inappropriate drug or spectrum, overlooking contraindications or dose adjustment, or failing to consider allergies, drug interactions, local resistance patterns, source control, and treatment duration.

Beyond response accuracy, clinical translation also depends on matching the role of LLMs to the complexity of the decision. Borgonovo et al. noted that LLMs may support evidence retrieval, guideline interpretation, and routine clinical queries [74]. As treatment becomes more individualized, their role should narrow because antimicrobial decisions must integrate patient factors, microbiology, source control, and local resistance patterns. In complex cases, LLMs may help organize information and identify missing evidence, but final treatment decisions should remain with clinicians. Conversational LLMs therefore remain promising but investigational tools requiring prospective validation and clinician oversight before routine clinical use.

The decision regarding optimal experimental methods and materials for AMR treatment requires researchers to review extensive publications, which poses a significant challenge. LLMs can serve as effective tools for literature mining, assisting researchers in identifying the most suitable methods and materials. Yang et al. fine-tuned ChatGPT using a carefully curated database to conduct literature mining in the field of self-assembling peptides. They began by automatically extracting section headings—such as material, method and experimental section—from PDF documents using computational tools. Relevant content was then collected based on keyword matching. Finally, the selected content was input into ChatGPT to summarize key features of the publications, achieving an average accuracy of 80% [75].

Beyond supporting the selection of specific methods and materials, AI-based literature mining can also reveal broader research gaps from a One Health perspective. Chen et al. applied AI-based NLP to analyze 254,738 AMR publications spanning two decades. Their analysis showed that limited intersectoral research and differences between culture-based and genomic methods hinder communication across One Health sectors. These findings underscore the need to apply both approaches in tandem across all sectors and strengthen research capacity in low-income countries to accelerate holistic One Health solutions to AMR [76].

### 5.3 The application of AI in One Health AMR surveillance and policy synthesis

AI-supported surveillance refers to the analysis of epidemiological, microbiological, genomic, and environmental data to detect resistance trends, identify hotspots, and forecast emerging risks. From a One Health perspective, AMR surveillance should integrate data across human, animal, food-production, wastewater, and environmental reservoirs. Lund et al. combined nearly one million bacterial genomes with more than 20,000 metagenomes across human, animal, soil, water, and wastewater microbiomes, using random forests to predict patterns of horizontal ARG dissemination [77]. Baker et al. integrated metagenomic and phenotypic data from chickens, carcasses, farms, abattoirs, and associated environments to identify shared potentially mobile ARGs and features associated with *Escherichia coli* resistance profiles [78]. At the global scale, Zhao et al. combined animal AMR surveys with environmental, antimicrobial-use, and livestock-density data to predict spatial resistance hotspots and antimicrobial classes likely to exceed critical resistance thresholds [79]. These studies demonstrate the potential of AI to integrate cross-sector data and support targeted surveillance and early risk detection, but they do not yet represent fully integrated human–animal–environment surveillance systems.

The performance and generalizability of AI-based surveillance systems ultimately depend on the availability of high-quality, standardized, and longitudinal surveillance data. This limitation highlights the importance of surveillance infrastructure. Pennisi et al. conducted four standardized annual point-prevalence surveys between 2022 and 2025, capturing healthcare-associated infection prevalence, microbiological profiles, AMR patterns, and patient risk factors [80]. It illustrates that repeated, structured epidemiological data can capture temporal changes and support AMR trend detection and risk forecasting. Therefore, AI-supported AMR surveillance requires not only advanced analytical approaches but also reliable and sustained data-generation systems.

Unlike surveillance systems that analyze epidemiological and microbiological data, conversational LLMs can support AMR policy development by integrating guidelines and policy documents from different regions. To advance One Health goals, AI-assisted systems could further connect recommendations on antimicrobial use in human and veterinary medicine with data from farms, food-production systems, wastewater, and environmental surveillance. Such tools may be particularly valuable where limited funding and uneven development constrain access to comprehensive policy evidence.

Chen et al. developed AMR-Policy GPT, a RAG-based framework using a multilingual database of 383 AMR policy documents from 146 countries [81]. The system combines BGE-M3 retrieval, semantic reranking, and GPT-4 to provide traceable references and supports both document-based and hybrid response modes. Validation demonstrated 87.3% accuracy in financial-plan extraction and 93.1% efficacy for multilingual queries. AMR-Policy GPT establishes a scalable template for evidence-driven policy assistance across global health domains.

However, RAG cannot eliminate hallucinations. It may retrieve irrelevant or outdated sources, misread conflicting guidelines, or combine accurate evidence into an incorrect answer. Therefore, RAG-based systems should support source traceability and citation verification, while their knowledge bases require regular updating. They should also communicate uncertainty clearly and abstain from definitive recommendations when the available evidence is insufficient or conflicting.

## 6 Outlook

Although AI-based research in biology has grown rapidly, few approaches have successfully translated into clinical practice. Looking ahead, a major opportunity lies in using AI to integrate the rapidly expanding multimodal data generated across microbial and host systems. These data include genomics, transcriptomics, bacterial single-cell RNA sequencing, proteomics, and microbiome profiles, which traditional statistical methods often struggle to analyze collectively. By enabling large-scale data processing and multimodal learning, AI may help clarify incompletely understood resistance mechanisms and identify meaningful therapeutic targets. The distinctive contribution of this review is its cross-scale perspective, which emphasizes integrating multimodal data across translational stages. Foundation models and language-model-based approaches may facilitate this integration, but their translational value will depend on prospective validation, interoperable data systems, and governance mechanisms that preserve accountability across settings.

High-quality, comprehensive, and representative databases are fundamental to the development and clinical translation of AI for AMR. Expanding and diversifying resources such as ARG databases, chemical libraries, bacteriophage datasets and clinical datasets is therefore essential. However, increasing database size alone is insufficient, because unrepresentative training data can introduce algorithmic bias and undermine model generalizability. Models developed in restricted populations may perform inconsistently across age groups, healthcare settings, and geographic regions, highlighting the need for subgroup-specific validation. This issue is particularly important for vulnerable populations such as older adults, in whom frailty, multimorbidity, polypharmacy, and atypical presentations may influence diagnosis, prognosis, and treatment decisions [82]. Geographical representativeness and equity are equally important for the global implementation of AI in AMR. Many genomic, clinical, and surveillance datasets are concentrated in well-resourced settings, potentially limiting model performance in low- and middle-income countries (LMICs) [83]. Implementation in these settings may be further constrained by limited laboratory capacity, digital infrastructure, and computational resources. Addressing these inequities will require locally led capacity building, affordable access to technology, and research partnerships that place LMIC scientists in leadership roles [76], [84].

Beyond data quality and representativeness, transparent model development is essential for the clinical translation of AI in AMR. Despite strong benchmark performance, many AI models remain “black boxes” whose opaque decision-making processes limit their reliability in real-world settings. Studies combining biochemical screening with metabolic network modeling illustrate how interpretable approaches can help elucidate antibiotic mechanisms [20]. Future studies should therefore prioritize explainable or intrinsically interpretable “white box” models that make their reasoning transparent to clinicians and regulators. Model interpretability, however, should be distinguished from post-hoc explanation. Interpretable models reveal how predictions are generated, whereas post-hoc methods attempt to explain predictions after they have been produced [85]. Post-hoc explanations may not faithfully represent a model's internal reasoning and may create unwarranted confidence without improving decision quality [86], [87]. Such explanations should therefore be evaluated according to whether they improve transparency, facilitate error detection, and support appropriate clinical use.

Reliable clinical translation requires AMR predictive models to undergo rigorous evaluation aligned with their intended use. Internal validation should assess discrimination, calibration, and classification performance while accounting for model overfitting. In imbalanced datasets, accuracy alone can be misleading; complementary measures should include AUROC, sensitivity, specificity, predictive values, precision–recall performance, and calibration. External validation across populations, healthcare institutions, geographic regions, and time periods is needed to establish generalizability, while decision-curve analysis can assess clinical utility. Importantly, the choice of evaluation criteria should reflect the intended application. Rule-out screening models may prioritize sensitivity and negative predictive value [88], whereas therapy-support systems require reliable resistance prediction without promoting unnecessary broad-spectrum treatment [89]. However, many studies reviewed here emphasized accuracy, sensitivity, and specificity, with limited assessment of calibration, external validation, or clinical utility. Future studies should therefore adopt evaluation frameworks that integrate discrimination, calibration, generalizability, and real-world impact.

In parallel, wet-lab experiments remain critical for validating antimicrobial activity and clarifying host–microbe and phage–bacteria interactions. For AI-assisted antimicrobial discovery, computational generation is only the entry point to translation. AI-generated candidates should advance from computational prediction to animal studies evaluating efficacy and safety, followed by clinical trials establishing therapeutic benefit. Clinical AI systems should likewise advance from computational and external validation to prospective evaluation and implementation studies that demonstrate patient- or population-level benefit.

At the implementation stage, AI-based rapid diagnostics and resistance-prediction tools should be integrated into antimicrobial stewardship programs rather than deployed as stand-alone systems. Such integration may help patients receive appropriate treatment sooner while avoiding unnecessary antibiotic exposure. Evidence from bloodstream infections supports this approach, as rapid diagnostics shortened the time to optimal therapy more effectively when combined with stewardship than when used alone [90]. AI should therefore be judged by whether it improves prescribing and patient care in accordance with local guidance and WHO AWaRe principles, rather than by predictive accuracy alone.

Clinical implementation must be accompanied by continuous lifecycle monitoring and governance. Because resistance epidemiology evolves, comprehensive databases available at deployment are insufficient; training and validation data must be updated over time. A prospective validation of MALDI-TOF-based AMR classifiers, for example, identified declining performance across several pathogen–antimicrobial pairs within 18 months [91]. Post-deployment monitoring should therefore detect changes in input data, calibration, and subgroup performance, triggering recalibration or retraining when necessary. Governance must also address the privacy and cybersecurity risks associated with large-scale omics data. Robust oversight should protect sensitive data, ensure traceability and accountability, regulate model updates, and preserve human responsibility for clinical decisions.

Looking ahead, an expanding range of novel approaches and emerging technologies could be harnessed to combat AMR. ML analysis of bacterial volatile organic compounds may provide diagnostic indicators, while AI-driven digital twins could help predict treatment toxicity and host responses. AI could also improve precision antimicrobial delivery by optimizing delivery materials and surface modifications, thereby limiting systemic drug exposure and potentially reducing selection for resistance.

AI could advance One Health approaches to AMR by integrating multimodal data from human, animal, food, and environmental sources. However, as highlighted by previous studies, research and data remain fragmented across sectors [76]. Differences in data generation and surveillance practices further limit their comparability and integration into unified AI frameworks. Future work should standardize data collection across sectors, track changes over time, and test findings in different settings. This could help One Health programs distinguish meaningful cross-sector links from coincidental associations and identify where transmission is most likely to occur.

Greater attention must be given to AMR as a critical global health challenge. AI may serve as a valuable tool in addressing it, but its impact will depend on robust validation, effective clinical integration, and appropriate governance.

## CRediT authorship contribution statement

**Qi Xia:** Writing – review & editing, Writing – original draft, Visualization, Conceptualization. **Jiayang Li:** Writing – review & editing, Writing – original draft, Conceptualization. **Wenqi Wu:** Writing – review & editing, Validation. **Jiajie Wang:** Writing – review & editing, Validation. **Zhitao Zhou:** Writing – review & editing, Validation. **Meilin Wu:** Writing – review & editing, Validation. **Mingjie Qiu:** Writing – review & editing, Validation. **Li Xu:** Writing – review & editing, Validation. **Jianan Ren:** Writing – review & editing, Validation, Supervision. **Xiuwen Wu:** Writing – review & editing, Supervision, Funding acquisition, Conceptualization.

## Ethical approval

Not required.

## Funding

We declare no competing interests. This work was supported by the 10.13039/501100001809National Natural Science Foundation of China (82272237) and the 10.13039/501100010035Outstanding Youth Foundation of Jiangsu Province of China (BK20250108).

## Declaration of competing interest

None declared.

## Data Availability

Not applicable.
